# Increased family psychosocial focus during children’s developmental assessments: a study of parents’ views

**DOI:** 10.1186/s12887-024-04800-4

**Published:** 2024-05-15

**Authors:** Sarah Strøyer de Voss, Philip Michael John Wilson, Ruth Kirk Ertmann, Gritt Overbeck

**Affiliations:** Centre for General Practice, Department of Public Health, University of Copenhagen, Øster Farimagsgade 5, bg. 24, opg. Q, København K, 1353 Denmark

**Keywords:** Psychosocial, Wellbeing, Mental health, Infant mental health, Child health, Preventive care, Developmental assessments

## Abstract

**Background:**

Family psychosocial challenges during the early years of a child’s life are associated with later mental and physical health problems for the child. An increased psychosocial focus on parents in routine child developmental assessments may therefore be justified.

**Methods:**

Participants in this qualitative study included 11 mothers and one parental couple (mother and father) with children aged 9–23 months. Participants were recruited to Project Family Wellbeing through their general practice in Denmark. Twelve interviews were conducted, transcribed and analysed with a deductive approach. The topic guide drew on the core components of the Health Belief Model, which also served as a framework for the coding that was conducted using thematic analysis.

**Results:**

Results are presented in four themes and 11 subthemes in total. Parents welcome discussion of their psychosocial circumstances during their child’s developmental assessments. Clinicians’ initiatives to address psychosocial challenges and alignment of parents’ and clinicians’ expectations may be required to allow this discussion. A flowing conversation, an open communication style and a trustful relationship facilitate psychosocial discussion. Barriers included short consultation time, concerns about how information was used and when parents found specific psychosocial aspects stigmatising or irrelevant to discuss.

**Conclusion:**

Enquiry about the family’s psychosocial circumstances in routine developmental assessments is acceptable among parents. Alignment of clinical and parental expectations of developmental assessments could facilitate the process. Future research should examine the predictive validity of the various components of developmental assessments.

**Trial registration:**

This is a qualitative study. The study participants are part of the cohort from Project Family Wellbeing (FamilieTrivsel). The project’s trial registry number: NCT04129359. Registered October 16th 2019.

**Supplementary Information:**

The online version contains supplementary material available at 10.1186/s12887-024-04800-4.

## Background

Infancy and early childhood are vulnerable periods of brain development [1], and strong indicators of risk linked to brain development are evident by age three years [2]. Multiple risks and resilience factors affect infant mental health, and deviant behaviour in infants and young children is associated with psychopathology later in life [1, 3]. Thus, identifying and addressing health risk factors in infancy and early childhood may avert future physical and mental health problems [4–6]. Parent-child interaction and relationships are the most important factors affecting infant mental health; low parental sensitivity (responsiveness) and insecure or disorganised attachment constitute significant risk factors, whereas sensitive parental behaviour and secure attachment serve as protective factors [1, 7–10]. Parental behaviour is in turn affected by parents’ psychosocial circumstances [1, 11, 12]. These include poor mental health [11, 13–16], poor general health, split homes [17], low income [16], more than three children in the home, multiple moves [11], domestic violence [13, 16], lack of social support [13] and low maternal education [14]. Furthermore, economic stress and relationship stress can directly influence maternal depression [9]. Addressing parental psychosocial functioning may therefore be important when aiming to detect and mitigate risk factors in infancy and early childhood and improve future health in children [1, 18–20].

Many healthcare systems offer developmental assessments to monitor child development and to identify preventable health problems early [21–24] by so-called universal prevention [1]. Developmental assessments are offered in different settings [25], and provide an opportunity to detect risk factors and potentially refer to relevant interventions [18–20, 24, 26]. The approach to preventive developmental assessments is, however, heavily influenced by cultural factors affecting both the process of the assessments and the expectations held by healthcare professionals and caregivers regarding the clinical focus [22, 23, 25]. Traditionally, developmental assessments have their main focus on the physical examination [26–28], and assessment of the child’s environment including parental psychosocial circumstances has never gained equal status with physical assessment [29, 30]. This is reflected in the inconsistency in which clinicians assess psychosocial factors and in the parents’ expectations of the developmental assessments [29, 30].

We have previously examined clinicians’ views on having an increased family psychosocial focus during the developmental assessments reinforced by use of structured child records [31]. While the clinicians usually had a systematic approach to addressing and examining physical development, it was novel to approach parents’ psychosocial circumstances systematically. Through use of the structured child records, clinicians gained an increased psychosocial focus, which improved their knowledge of the families, strengthened clinician-parent relationship and helped uncover psychosocial challenges early in the child’s life. Addressing family psychosocial circumstances did sometimes raise feelings of discomfort in the clinicians, especially when addressing sensitive matters not expected by the parents or if clinicians did not have a solution to the parents’ psychosocial challenges [31]. Clinicians considered that the reasoning behind addressing parental psychosocial aspects might not be obvious to parents attending developmental assessments with their children. Thus, there is a need to explore parental views on an increased psychosocial focus in the developmental assessments to discover whether parents find it meaningful and acceptable, and to establish their views on how these topics can be covered.

Based on the study of clinicians’ experiences with the structured child records with increased psychosocial focus [31], we generated the hypothesis that most parents would not initiate discussion of their psychosocial circumstances during their child’s development assessments. The Health Belief Model was found relevant in shedding light on factors important to changing parental perspectives on psychosocial discussions at these assessments [32]. The Health Belief Model has previously been used to examine parental behaviour towards their children, for example in relation to acceptance of vaccines [33]. It builds on the idea that in order to be motivated to change behaviour, there should be a potential treat (perceived susceptibility and severity) and the benefits of changing behaviour should outweigh the burdens [32]. Often cues to action can be identified, in the form of factors facilitating the change of behaviour [32].

The current study investigates parental perspectives on the implementation of developmental assessments with an increased psychosocial focus, aided by structured child records within Danish general practice. The aim of this study is to explore parents’ experiences of these child developmental assessments, which include discussion of the family’s psychosocial circumstances within the consultation.

## Methods

### Study design

This study is based on 12 interviews with 11 mothers and one couple (a mother and a father), who all had children aged between 9 and 23 months and who had attended general practice for routine developmental assessments. Interviews were conducted by SV in a semi-structured manner to ensure coverage of relevant topics while affording opportunities for participants to bring up related topics. The Health Belief Model inspired the topic guide [32], including factors that affect motivation for a specific behaviour preventing a negative health outcome – in this case discussing psychosocial aspects with their clinician during their child’s developmental assessment. According to the Health Belief Model, parents should view psychosocial challenges as a potential threat to their child’s health, equivalent to physical problems (susceptibility and severity). In addition, it proposes the benefits of discussing these aspects with the clinician should outweigh the disadvantages (barriers) of not discussing it [32]. Sample questions include: “Please try to describe how you experienced the developmental assessment from beginning to end”, “what was different, if anything, compared to your expectations?”, “what do you find appropriate to discuss at the developmental assessment?”, “what could encourage you to open up about personal aspects?” (See ‘Additional file 1: topic guide’).

### Context

This interview study was nested in Project Family Wellbeing, a cluster-randomised trial testing the effect of a web-based mentalization program on children’s social skills and language development [34]. The project involves 650 families from pregnancy to child age 31 months. The clinicians (GPs, nurses, midwives) conducting the developmental assessments in the trial had attended a one-day course on delivering these assessments in structured format with an increased psychosocial focus compared to existing standard assessment protocols. The structured child records served to prompt and record information gathered by clinicians through the course of routine developmental assessments. The use of structured child records leads to conversation about the family’s mental health, social network, relationships, socioeconomic factors as well as the child’s physical and cognitive development, milestones etc. It also contains an observational assessment of the parent-child interaction (see ‘Additional file 2: example of a structured child record’). In Project Family Wellbeing, the clinicians were able to offer additional time in developmental assessments, but not all chose to do so. The clinicians were positive about the increased psychosocial focus gained by the use of the structured child records, however, some barriers led to some clinicians discontinuing their use [31].

### Setting

This study took place in Denmark, where all families with small children are offered seven developmental assessments, free of charge, in general practice within the child’s first five years of life (at 5 weeks, 5 months, 1, 2, 3, 4 and 5 years) [27]. The developmental assessments can by carried out by the GP, nurses or midwives working in general practice. The participation rate for the first three developmental assessments is 91–92%. The 2-year assessment has a lower participation rate at 63%, as it is not combined with vaccination [35]. All families are also offered five home visits from a community child health nurse (health visitor) within the child’s first 10 months [27].

### Data collection

The sample was drawn from families participating in Project Family Wellbeing [34]. The starting point of the interview was the experience of a specific developmental assessment that the parent(s) had attended with their child. Participants from both the intervention group (introduced to a web-based mentalization program) and the control group were included. To ensure variety in participants’ experiences and perspectives, the sample selection was first based on clinics where they were seen and secondly on participant characteristics. It was important that most participants had been exposed to the psychosocial focus imbedded in the structured child record. At the point of selection (February 1st 2022), 47 of 58 clinics appeared to be actively using the structured child records and 15 clinics had completed more than 10 structured child records at the 5 weeks developmental assessment. Thus, we assumed these were the clinics most active in adopting the novel assessment approach with increased psychosocial focus. Four of the clinics were in the centre of Copenhagen and only one of these was chosen – the only practice with a male general practitioner. The final sample was drawn from patients in 12 clinics: five in Region Zealand and seven in the Capital Region, five in the control and seven in the intervention group. The participants were invited to reflect diversity in age and parity (see ‘Table 1: participant characteristics’). We included mothers with a range of engagement with the web-based intervention in Project Family Wellbeing, which aimed to increase mentalization ability [34]. Participants who had participated in other interviews related to Project Family Wellbeing were excluded. The participants were recruited in three rounds to ensure ‘information power’ in the sample size [36]. During this process, a participant from each selected clinic and diversity in participant characteristics were prioritised. Eight invited mothers did not respond to the invitation, and a participant with similar characteristics replaced each non-responder. Ten mothers were recruited through a digital secure mailbox (e-boks) and two by phone call. Both parents were invited to join the interview but only the mother was contacted directly. One father was recruited through the mother.

From March to June 2022, nine interviews were conducted in the participants’ homes while the remaining three were conducted via video connection due to participants’ preferences. The interviewer and participants had no relationship prior to the interviews. Participants were informed that SV was a physician. All interviews were audio recorded and manually transcribed verbatim by SV. Personal data were pseudonymised during the transcription process. All participants gave informed consent to the recordings and use of data according to applicable regulations. Interviews lasted between 28 and 70 min (mean 48 min).

### Characteristics of participants

Eleven interviews were held with mothers and one interview was held with both the mother and the father. Participants’ children were between 9 and 23 months. Some participants were first-time parents (*n* = 7) while others had older children (*n* = 6). Eleven parents lived with their partner (the co-parent), and two mothers were single parents. Their age ranged from 25 to 47 years (mean = 32). They lived in a variety of areas; urban (*n* = 6), suburban (*n* = 5) and rural (*n* = 2). The parents were generally well educated, and all had jobs or were studying. All participants were white with Danish origin, and fathers were poorly represented. See Table 1 for distribution of participant characteristics.

**Table 1:** Participant characteristics

Interview	Sex	Age (years)	Area	Education level [37, 38]	Civil status	Number of children	Age of the index child (months)
1	F	25–29	Suburban	Higher national diploma* (level 5)	Married	2	22
2	F	35–39	Urban	Bachelor degree (level 6)	Married	2	10
3	F	25–29	Urban	Higher national certificate (level 4)	Married	3	16
4	F	45–49	Suburban	Master degree (level 7)	Married	2	20
5	F	30–34	Suburban	Bachelor degree (level 6)	Married	1	19
6	F	30–34	Urban	General certificate of secondary education (level 2)	Married	1	18
7	F	25–29	Urban	Bachelor degree* (level 6)	Cohabiting	1	9
7	M	25–29	Urban	Master degree (level 7)	Cohabiting	1	9
8	F	25–29	Suburban	Bachelor degree (level 6)	Cohabiting	3	10
9	F	25–29	Suburban	Bachelor degree* (level 6)	Cohabiting	1	18
10	F	40–44	Urban	Master degree (level 7)	Single	1	18
11	F	35–39	Rural	Master degree (level 7)	Single	1	18
12	F	30–34	Rural	Bachelor degree (level 6)	Cohabiting	2	23

*Currently studying for the indicated degree

### Data analysis

Analysis was conducted with an overall deductive approach with the Health Belief Model inspiring the topic guide prior to the coding process [32]. Six-phased thematic analysis was used during the coding process as described by Braun and Clarke: (1) familiarisation with the data, (2) initial coding, (3) creating themes, (4) revising themes, (5) finalising themes, and (6) reporting the findings [39]. Initial coding was based on important aspects addressed by the participants. Codes were grouped and later rearranged into preliminary subthemes inspired by the Health Belief Model [24]. This was done by SV. The final themes were agreed upon during an iterative process by continuous discussion between all authors [40] (see ‘Additional file 3: coding tree’).

## Results

In accordance with the Health Belief Model: The first theme ‘It is meaningful to discuss psychosocial aspects’ encompasses parental views on whether psychosocial challenges are relevant to a child’s wellbeing and whether there might be benefits in discussing these challenges with a clinician. The second theme ‘Parental concerns about discussing psychosocial challenges’ covers barriers to discuss psychosocial aspects with the clinician. The third theme ‘Clinicians are responsible for conversations about psychosocial aspects’ refers to cues to action related to initiating and leading a conversation about psychosocial aspects. The fourth and final theme ‘Good communication facilitates discussion of psychosocial challenges’ describes cues to action related to creating a foundation for good communication about psychosocial aspects. These themes shed light on how psychosocial factors are addressed during the developmental assessments and how psychosocial discussion could be facilitated.

### Theme 1: it is meaningful to discuss psychosocial aspects

#### Psychosocial challenges are common experiences for families

Most of the parents had experienced some type of psychosocial challenges in parenthood; feeling overwhelmed, exhausted, stressed, experiencing anxiety, post-partum depression or the new family structure took a toll on the parents’ relationship with each other. According to most parents, relationships between parents, problems with siblings and social networks could easily be affected when receiving a new child into the family:

*“I think something like the relationship between the parents and how they are doing, because it’s a big transition to become parents, even for a second time. I think it has been a radical change in our everyday life.”*

- Mother 2.

Most parents also described family and friends experiencing these types of challenges when becoming parents.

#### The overall wellbeing of the family is viewed as important to the child

All the parents considered the family’s overall wellbeing along with regulation of the child to be important to the child’s health and development. Everyone particularly viewed parental mental ill-health as important, because this could easily be affected during the transition to parenthood, for example feeling stressed or overwhelmed, having anxiety, and experiencing low mood or depression:

*“It’s of course a radical change to have a child, and you can have many questions and many feelings (…) it would be very nice, that every time you meet a health care professional there is someone who is aware how you are doing even though in theory it’s not the parents who are in for a check-up, it’s the child.”*

- Mother 9.

All the participants believed that parental mental ill-health could affect their child’s wellbeing, and several had experienced their mood directly affecting their child:

*“There is also a symbiosis between me and her. If I don’t feel good, then she doesn’t feel good either. No matter if she is doing well physically, then she can feel whether I don’t feel good.”*

- Mother 10.

Another mother described that a bad mood would ‘make her fuse shorter and make it easier for her to snap at the child’. Several respondents pointed out that their own wellbeing was particularly important after becoming a parent, because a child was now dependent on them. The impact of parental relationships, of problems with siblings and social networks were also considered of great importance as it could affect parental wellbeing and thus indirectly the wellbeing of the child.

#### Parents believe that clinicians have something valuable to contribute

While clinicians’ roles were well defined regarding physical problems, they appeared less clear in relation to psychosocial aspects. The parents had differing views on the clinicians’ roles regarding detection of problems. Most thought clinicians might be able to identify something in relation to the child’s development, however, a few parents were confident, they could detect the problems themselves:

*“It may be good that there is someone else [professionals] who can catch if the development is not how it’s supposed to be (…). But I don’t think he [the GP] could catch something regarding development, that I wouldn’t have caught myself first.”*

- Mother 2.

In general, the parents supported an increased focus on parental mental health and social challenges related to the life changes following childbirth:

*“It could be really good if there was more focus on it [parental wellbeing], from the doctor’s point of view as well. Then something could be picked up earlier that might not have been discovered in institutions, like day-care or maybe even later in school. (…) if there is low wellbeing at home, then it’s best to catch it early so the child isn’t damaged.”*

- Mother 1.

To some parents, discussing psychosocial challenges was useful in itself; while others pointed out that, it only made sense to discuss psychosocial challenges if it led to some type of help. The parents thought that clinicians played an important role in handling most types of psychosocial challenges. The importance of receiving advice and guidance related to psychosocial challenges was emphasised by several parents. Getting check-ups, receiving follow-ups or referrals were also viewed as essential. Most parents mentioned reassurance and praise as important elements of the developmental assessments, confirming the parents in their parental role and giving them confidence:

*“He [the GP] can give advice, guidance and be like ‘take it easy, relax’ and ‘it will be alright’, and like give you reassurance that you are doing a good enough job and they [the children] are healthy and well.”*

- Mother 8.

Some parents viewed the developmental assessments as a place to get something off their chest, and they found it meaningful to discuss their concerns with a professional. Other parents, however, viewed the health visitor as a more appropriate professional to turn to when experiencing frustrations related to parenthood:

*“Well it’s hard to be a mother, but I might not talk to my GP about those things. I would probably talk to the health visitor instead. You know, things like: ‘she isn’t sleeping’, ‘she is crying’ that type of thing. I think for the doctor it would be more like (…) you know, some things, you think a doctor should assess like: ‘does this look right?’”*

- Mother 9.

Seeking help from psychologists or social services for specific problems were mentioned as alternatives.

### Theme 2: parental concerns about discussing psychosocial challenges

#### If time seems limited then parents are reluctant to discuss psychosocial aspects

Several parents highlighted the additional time as very positive, and they emphasised the importance of having sufficient time if they were to open up about psychosocial aspects.

Some parents were very aware of the fact that they were using the clinician’s time, from which they did not want to “steal” too much, because they believed the clinicians to be very busy. Consequently, some did not feel there was enough time to discuss the family’s wellbeing:

*“There is a set amount of time and it feels like there is a kind of a plan (…). There are some things that she [the doctor] has to cover and record, and I don’t actually feel there is time for that [to talk about parents’ wellbeing].”*

- Mother 4.

Some feared opening up about sensitive topics as if it could represent a ‘Pandora’s Box’, so they avoided going into details when asked questions about psychosocial aspects:

*“If (…) you have to be out the door again – you wouldn’t open up about something if you didn’t feel there was enough time to shut it down again.”*

- Mother 2.

#### Some psychosocial aspects were found irrelevant in this context

##### Education, income and housing

The parents did not see all psychosocial aspects as equally important. Questions regarding education, income and housing were thought inappropriate by some parents unless it was related to something they had mentioned themselves. They expressed concern about a risk that parents would feel stigmatised if they had no education, low income or a small home.

*“I think that’s a question that oversteps boundaries a bit, if someone has to assess if we have capacity for a child to come (…) whether economically or emotionally.”*

- Mother 9.

Some would be reluctant to answer questions of this type, while others would answer but would find it strange. Furthermore, several parents also found education, income and housing irrelevant to the overall wellbeing of the parents, and they emphasised that this was not necessarily related to whether a child was happy and well-functioning. On the other hand, if the parents were stressed or had anxiety because of their finances or accommodation then it would be appropriate to discuss:

*“I just think, for instance, if a mum and dad are fighting or are very upset or angry or they have bad finances, they might act more distractedly and are not present because they have other things on their mind. I definitely think it can remove focus from the child and affect it negatively.”*

- Mother 8.

Thus, it all depended on the context and how the questions were asked. Only a few had actually been asked questions related to these aspects, but parents who experienced discussion about education or housing found it to be a natural part of the conversation:

*“I didn’t find it inappropriate that she [the GP] asked about that [accommodation]. To me, it just showed that she had a sincere interest in her patients – how their living conditions are, right? And I think, I would have felt the same (…) if she had asked me about my finances – then I don’t think that would have overstepped my boundaries either.”*

- Mother 3.

##### Sex life and religion

A few thought it was inappropriate to discuss their sex life. Some found this to be too personal while others just thought it was inappropriate in the context. Most found it inappropriate if the child was more than one year of age. A mother also mentioned religion as an inappropriate topic as this was considered a private matter and she had previously experienced feeling stigmatised regarding having her child circumcised due to religious beliefs.

#### Concerns about how the information is used

All parents found it important that clinicians disclosed why they asked about certain psychosocial aspects and how the information was used. They suggested that the clinicians should be clear about when they were making small talk and when something was going into the record. Parents also wanted to know why things were recorded, for instance to follow up at the next consultation, to make a referral or a report to other agencies.

*“I would answer, but again it would be hard for me to understand what she [the doctor] wanted it for or how she would use it, and then I might be a little concerned, if she wanted to notify social services (…). I would find it odd if someone asked me about accommodation and finances and all these types of things without me knowing how this information is used.”*

- Mother 12.

A few parents mentioned that certain question could trigger a fear of being reported to social services. If parents felt unsure about how the information was used or if the clinician asked about something sensitive without the necessary safeguards the parents might avoid the conversation or feel like defending themselves. Many parents also pointed out that they did not have particularly sensitive or severe problems themselves and it might be more difficult to discuss psychosocial aspects if they were vulnerable or had severe problems:

*“In general, it’s probably more overstepping the boundary to be asked about things where you are vulnerable.”*

- Mother 7.

### Theme 3: clinicians are responsible for conversations about psychosocial aspects

#### Parents want expectation alignment

All parents had expectations about their children being weighed and measured and assessed physically during the developmental assessment. Most of them also expected that they could bring up concerns about their child’s development and behaviour. Few expected to address psychosocial aspects regarding their family. Many parents would have liked an expectation alignment in advance to prepare them for psychosocial aspects being addressed. That way, they would not feel singled out for discussion of these issues:

*“(…) because ‘okay, is that what I’m projecting? That something isn’t right at home?’ Because you go there and maybe you have not showered for three days and you might have porridge in your hair (…) so you might feel a little on the spot if you are suddenly being asked about something you didn’t expect at these child developmental assessments.”*

- Mother 1.

If the clinician brought up challenges that the parents did not expect and they felt on the spot, many would answer briefly and shut down the conversation:

*“It might be that I would automatically shut down the conversation, because it [family relations and parenting] was not what I expected to be going into.”*

- Father 7.

Some parents thought that being informed about the agenda prior to the developmental assessments would give them a chance to consider their own needs for discussing specific psychosocial aspects with the clinician:

*“For me it would be good. I would be able to just write down some of the things I am thinking during the week, and then there would be something concrete to come back to [during the assessment].”*

- Mother 7.

The parents were presented with the idea of using a questionnaire prior to the developmental assessment in order to align expectations and to use as a tool for steering the conversation during the consultation. Some were positive towards this idea especially if sent to them digitally days in advance thereby giving them a chance to reflect on their needs. Others did not want to allocate time to this. A few pointed out that parents might not be completely honest when filling out questionnaires if there appeared to be a positive and a negative answer.

#### Clinicians should lead the conversation about psychosocial aspects

Because most parents did not expect to talk about psychosocial aspects, they considered it important that the clinician should initiate this conversation:

*“Well, I would never go in and just like, open myself up. There would have to be some questions asked for me to do that.”*

- Mother 6.

Even though all parents would like to be able to discuss psychosocial challenges, some found it intimidating to initiate the conversation about their own mental health, especially if they did not have a close relationship with their clinician:

*“I would find it difficult to say: ‘by the way, things are not going well at home’. I don’t think I would find it appropriate.”*

- Mother 1.

Some parents needed clarifying questions from the clinician to take the conversation to a deeper level as they sometimes found it difficult to distinguish small talk from history taking. For instance, if the clinician asked: “how are thing at home?” They would tend to answer: “it’s fine” as the question would be simply too broad and if they were to answer honestly, they might have to make a long list of difficulties. All parents wanted the clinician to steer the conversation to make sure the conversation stayed on track, while still leaving room for them to bring up concerns.

### Theme 4: good communication facilitates discussion of psychosocial challenges

#### Continuity is important for the communication

Most parents found continuity and an established relationship with the clinician to be a facilitator when discussing psychosocial aspects:

*“I don’t think I would turn to my doctor if we had problems at home that were difficult to handle and affected us mentally (…) because turning to someone you don’t know really oversteps the boundaries as we don’t have a relationship with her apart from her looking at our wounds and injuries and stuff like that.”*

- Mother 1.

A few were, however, willing to discuss these issues regardless of prior knowledge of the clinician.

#### The clinician’s attitude is important

The attitude of the clinician was considered important by all the parents:

*“Well, it’s obviously also her [the GP’s] demeanour that has a lot to do with whether this is someone you want to talk to. If it wasn’t someone who I thought fitted me personally, then I think I would change doctor, because I want someone who is easy to approach and whom I can talk to.”*

- Mother 5.

If the clinician had a negative attitude or the parents felt judged, they would abstain from discussing psychosocial aspects. Sometimes the fear of being judged could prevent parents from going into certain conversations:

*“It could very easily prevent me from participating [in the conversation] if I was afraid of being judged for having problems that were stigmatised.”*

- Mother 7.

The parents emphasised the importance of the clinician being friendly, open-minded and sincerely interested. It was also very important to the parents that the clinicians took the parents’ concerns seriously in order for them to have a good and trustworthy alliance.

#### A flowing conversation makes it easier to open up

Most of the parents had experienced clinicians addressing some of the topics from the structured child records, but without being asked questions in a questionnaire-like manner. This was considered very important, as a questionnaire-like structure would inhibit a flowing conversation and make the clinician seem less sincere:

*“If it becomes too structured, I’ll probably back off a bit. I definitely think you have to look at the individual child, the individual parent and the individual family and adjust the questions to that (…). I am definitely more into a flowing conversation, which also makes my answers longer; there is no doubt about that.”*

- Mother 11.

A flowing conversation was thought to be important in order not to feel that there were any right or wrong answers, encouraging an honest discussion. Particularly when related to psychosocial challenges, the parents felt a flowing conversation was important, as issues in these areas are often more sensitive:

*“If the doctor just asks a lot of questions about a lot of other things aside from health, it might feel a little like an attack or feel like you’re being judged or you might feel you not doing a good job – it feels too overwhelming.”*

- Mother 3.

## Discussion

Parents found it meaningful to discuss family psychosocial functioning with the clinician during their child’s developmental assessments, since they believed that the wellbeing of the family influences the wellbeing and development of the child. Despite various backgrounds, most participants had experienced psychosocial challenges in relation to becoming parents. Overall, they believed that the clinician could help the family’s psychosocial challenges e.g. by giving advice or making referrals. Barriers to disclosure by parents of their psychosocial circumstances included time pressure, stigmatising questions and lack of knowledge about how the information would be used. Positive actions included the clinician leading the conversation about psychosocial aspects along with attention to their communication style. Furthermore, parents found potential for improved expectation alignment to facilitate discussion about psychosocial aspects.

In developmental assessments, physical examinations are known to be expected and accepted by parents in line with findings of this study [31, 41, 42]. Parents have emphasised that developmental assessments also constitute a unique opportunity to discuss their child’s development along with their own concerns [41]. Parental concerns have proven an important indicator for abnormal child development and behaviour [43–46], however, concerns regarding child behaviour and development are often overlooked or not handled adequately [46–48]. Furthermore, only a third of parents voice their concerns about their child to the GP [46]. In line with findings of this study, parents have previously addressed the importance of clinicians initiating conversation about topics outside the physical focus [41].

In accordance with our findings, child development and behaviour are culturally accepted topics during developmental assessments [29, 30, 41, 42], while psychosocial aspects related to the family are often viewed as more sensitive [29] and difficult to identify [49]. Postnatal depression screening might offer a useful comparison. A systematic review investigating its implementation found that women “might feel anxious and reluctant to answer questions honestly” [50](p. 338). As in our study, expectation alignment was an important factor when introducing a sensitive topic: if the mothers were informed about the content and purpose in advance, the questions would be more acceptable [50]. Maternal mental health was a topic that mothers in this study found very appropriate to discuss during the developmental assessments. This might relate to the fact that health visitors offer all Danish mothers postnatal depression screening, and therefore it would not be novel to them to discuss their mental health with a clinician. Expectations influence satisfaction with clinical encounters [51], thus use of a pre-consultation questionnaire can facilitate discussion of concerns, improve time management, increase support and patient satisfaction [52]. Furthermore, patients’ concerns identified with pre-consultation questionnaires are often not expected by clinicians [53]. Similarly, questionnaires have been suggested prior to developmental assessments to help align expectations and manage time [31, 41, 42]. The results of this study indicate that parents have mixed feelings about this idea. It could pose problems if parents felt the questions off-putting or stigmatising or if they did not answer honestly [51]. In such a case, the developmental assessment could get off to a bad start. Pre-consultation information or expectation alignment without a questionnaire could potentially improve the acceptability of discussing psychosocial aspects.

In line with our findings, previous studies have also pointed to the need for extended consultation time when addressing psychosocial factors [30, 31, 41, 47, 54]. Pre-consultation preparation might improve time management, but an increased psychosocial focus in the developmental assessments might require less time to be spent on other aspects like physical examination.

Communication was identified as an important theme and interpersonal communication theories might be more useful to shed light on how to change parental communication behaviour [55]. Shared decision-making plays an important role, thus talking about psychosocial aspects during developmental assessments has to be negotiated between parents and clinicians while respecting parental autonomy and preferences [55]. Clinicians having an open-minded attitude and interested communication style as well as parental trust in their clinician are other important factors that influence the acceptability of addressing psychosocial factors [29, 30, 55–58]. Disclosing private information involves boundaries regarding whom the information is shared with and mechanisms to protect the information from outsiders [59]. Seen in this light, it is understandable that parents have concerns when disclosing psychosocial aspects and about how their information is used. Furthermore, parents prefer a flowing conversation about psychosocial aspects, giving them opportunities to explain and ask clarifying questions [50]. Addressing psychosocial aspects in a flowing conversation contribute to the clinician appearing sincere and interested. It reduces the fear of being judged and makes it easier to be honest [50].

While developmental assessments are widely used in many western countries, there is variation in where they occur and who conducts them [22–25, 60]. This could lead to a debate about what is most efficient or whom parents prefer to turn to with concerns about their children. Parents with children who previously experienced health problems might primarily consult their GP regarding their child’s behaviour, while young mothers tend to use their health visitor to discuss their child’s behaviour [61]. Some mothers at risk felt especially vulnerable when a health professional was visiting their home [56, 58], which can be a barrier for discussing psychosocial challenges. Others have suggested that psychologists would be better equipped to discuss psychosocial challenges [49, 62], but it is unlikely that they could provide a universally accessible service. Preferences differ and may depend on previous experiences, established relationships and ‘chemistry’ with the clinician [56, 58, 61]. The different types of clinicians have different attributes regarding time frame, continuity, knowledge of family history, facility to visit family homes, expertise etc. Most important, collaboration and cross-referral can play an important role in assessing and handling children’s psychosocial wellbeing [30, 62, 63].

The paternal perspective is largely absent from the current paper as only one father was included. We know from to the literature that paternal mental ill-health can lead to adverse child outcomes by a combination of pathways, modified by child characteristics and parental psychosocial factors [64]. Fathers tend to seek support related to parenting and mental health among their social network or they may seek informational support online [65, 66]. Some turn to their GP when mental health problems become severe [65, 67]. Formal support from paternal groups is rarely accessible but may be desired by fathers [65, 66, 68]. Some fathers have previously emphasised the importance of addressing and normalising psychosocial challenges related to becoming a father [67, 69].

### Strengths and limitations

There were certain strengths to this study: During sampling, variety in socioeconomic status was sought along with variety in clinicians’ psychosocial focus was ensured by recruiting from both the intervention group and the control group of Project Family Wellbeing. All the participants had attended at least two developmental assessment in general practice prior to the interview.

The study also posed limitations: The fact that clinicians agreed to participate in Project Family Wellbeing could be associated with them having more interest in psychosocial aspects and mental health compared to clinicians outside the study. If this is the case, it is plausible that they were better at implementing the novel child assessments and discussing the family psychosocial environment compared to other clinicians, and that would affect parents’ experiences. At the same time, the families who agreed to participate in Project Family Wellbeing might have been more open to discussing psychosocial challenges compared to the wider population. In Denmark, the population in general has high living standards. It could have been useful to include vulnerable participants (or from low socioeconomic status), however, these could not be identified within the cohort.

Only mothers were recruited, except for one interview that was conducted with both parents including the father. More effort could have been put into illustrating the paternal perspective, but Project Family Wellbeing generally experienced challenges in recruiting fathers, which will be described in a separate paper.

The first author/interviewer’s (SV) former position as a doctor in general practice might have affected the participants’ answers [70]. All authors were, however, aware of this during analysis.

### Implications for practice

Checklist systems have been proposed to help increase the psychosocial focus in child consultations [20, 30, 62], in line with the purpose of the structured child records used in Project Family Wellbeing. The results of this study indicate that the structured child records allowed systematic data gathering in the consultation without parents experiencing it as a questionnaire-driven process. The actual questions were individualised and adjusted to the family and the situation allowing a flowing conversation as oppose to screening tools, which parents found important.

Aligning expectations prior to the developmental assessments could contribute to normalising psychosocial aspects being addressed in the consultation [31, 41, 50, 51]. This have recently been attempted with pre-consultation information to parents aiming to prepare them for psychosocial questions during the developmental assessment [71]. In Denmark, digital secure mails are sent to parents reminding them to schedule their child’s immunization. Similarly, an invitation to the developmental assessments could be sent to parents including an information letter about the purpose of the visit. It could also encourage parents to consider what they want to discuss and if they have any specific concerns.

Clinicians conducting developmental assessments need adequate training in interviewing and counselling techniques to be equipped to address disclosure of psychosocial challenges [31]. Explicitly addressing the presence of sensitive topics and setting the boundaries could ease parental concerns and show respect for patient autonomy [59]. Our findings align with the principles of the reciprocity rule in patient-clinician communication in which clinicians offer counseling or referrals in exchange for patients/parents sharing sensitive information [31, 59]. To effectively navigate these interactions, clinicians might benefit from training in brief counselling techniques and strategies to help families activate their resources and apply useful coping techniques [72].

### Implications for research

Evaluation of counselling techniques to assist clinicians in helping parents handle psychosocial challenges could be beneficial [31, 72]. In addition, creating closer collaboration between different actors (e.g. GPs, nurses, health visitors and social workers) could improve the way families’ psychosocial challenges are handled [30, 62, 63] Further research is needed to identify focus points and to test strategies in this area.

The implementation of structured child records with increased psychosocial focus should be investigated in other regions with similar health care systems. Other approaches to increase psychosocial focus in the developmental assessments should be examined as well. Besides evaluating clinicians’ and parents’ experiences, quantitative outcome measures could be included - for instance number of counselling sessions, referrals and diagnoses. Finally, more research in developmental assessments is needed to gather evidence of the predictive validity of the various elements (e.g. discussion points and tests) of developmental assessments.

## Conclusion

Family psychosocial wellbeing is crucial to children’s health and development. Parents acknowledge this and they appreciate discussion with the clinician at their child’s developmental assessments about most aspects of family psychosocial functioning. It is, however, important that expectations are aligned prior to the consultation and that the clinician initiates the conversation about psychosocial aspects. A checklist can assist clinicians to provide a systematic approach as long as the conversation remains flowing. An open communication style and a trusting relationship play a key part in allowing clinicians to address sensitive topics. Communication training and collaboration between GPs and other actors should be prioritised. Future research should investigate the implementation of structured child records in other regions and should include quantitative process and outcome measures. Most importantly, evidence for the relative value of the different elements of the developmental assessments, including the physical examination, is required.

## Research Reflexivity

Prior to the study, SV experienced that developmental assessments in general practice usually did not have much psychosocial focus. During her training in developmental assessments, the focus was mostly on the physical examination. SV went to the interviews with an exploratory approach. PW worked as a GP in rural and urban areas of Scotland and experienced removal of child health surveillance work from Scottish general practice in 2004. RE works as a GP in Denmark in an area with low socioeconomic status. She normally has a high focus on psychosocial aspects during the developmental assessments, and she was part of the group developing the structured child records. GO is a language psychologist. SV is conducting her Ph.D. while GO, RE and PW are all established researchers working within the field of child/family health. All authors have children. The authors’ different backgrounds and experiences with families and children (including their own) added many different perspectives to the discussions. Throughout analysis and write-up of this paper, the authors have been mindful that this paper should reflect the parental views on an increased psychosocial focus in the developmental assessments regardless of the literature or the authors’ clinical experiences.

### Electronic supplementary material

Below is the link to the electronic supplementary material.

Supplementary Material 1

Supplementary Material 2

Supplementary Material 3

## Data Availability

The dataset is not publicly available as it contains sensitive statements, which could potentially be used to identify the participants. In case of queries, please contact corresponding author, Sarah de Voss.

## Abbreviations

GP: General practitioner
GPs: General practitioners
