# Supplementary material for: Increased family psychosocial focus during children’s developmental assessments: a study of parents’ views
Source: BMC Pediatr. 2024 May 15;24:335. doi: 10.1186/s12887-024-04800-4 (PMC11094963; doi:10.1186/s12887-024-04800-4)
Supplement: Supplementary file 1 — Supplementary Material 1 [file 12887_2024_4800_MOESM1_ESM.pdf]

Additional file 1: topic guide

About parents' experience of the developmental assessments

This topic guide was translated from Danish to English after interviews were conducted.

**Introduction**	• Give information about the study.<br>• Introduction of the interviewer and her part in the study.<br>• Get oral consent to participation, recording and storage of data.
**Background**	• Would you tell me a little bit about you and your family?<br>(should cover age, education, job, family constellation)<br>• How were you introduced to Project Family Wellbeing?<br>• What thoughts did you initially have about the project when it was introduced to you?<br><br>• Who conducted your child's developmental assessments?<br>• How much contact did you have with him/her/them prior to the developmental assessments?<br>• How is your overall relationship with the clinician(s) who conducted the developmental assessments?
**Developmental assessment**	Try to think back to a specific child developmental assessment you have been to with your child that is part of the Family Wellbeing Project.<br><br>• Which developmental assessment are you thinking of?
**Expectations**	• Please try to describe your expectations prior to the developmental assessment.<br>• If older siblings: were these expectations different from when you were at your older child's developmental assessments?
**Experience**	• Please try to describe how you experienced the developmental assessment from beginning to end.<br>• What was different, if anything, compared to your expectations?<br>• If older siblings: was there anything different compared to developmental assessments of your older child?<br>• Was there anything in particular which the clinician helped you with?<br>• If yes: would you care to elaborate?<br>• Did you experience anything positive in the way the developmental assessment was conducted?

	• Did you experience anything negative in the way the developmental assessment was conducted?<br>• Did anything feel uncomfortable or to be overstepping your boundaries?<br>• If yes: would you care to elaborate?<br>• Could anything have been handled better?<br>• If yes: would you care to elaborate?<br>• What do you think about the questions that the clinician asked?<br>• Was there anything else you would have liked to discuss during the developmental assessment?<br>• If yes: would you care to elaborate?
**Health Belief Model**	• What do you find appropriate to talk to [clinician's name] about?<br>• What do you find appropriate to discuss at the developmental assessment?<br>• Are there any topics you would not feel like discussing at the developmental assessment?<br>• If yes: would you care to elaborate?<br>• Would you want to discuss concerns related to your mood, relationship with your partner or other family member, your finances or accommodation?<br>• Do you find these topics relevant to the developmental assessment?<br>• Do you think mental or social aspects in the family affect a young child?<br>• If yes: would you care to elaborate?<br>• What could encourage you to open up about personal aspects?<br>• What would inhibit you from discussing personal aspects?<br>• What kind of help do you think the clinician can provide if you bring up that type of personal problem?
**Closure**	• What is your overall thought on clinicians addressing mental and social aspects at the developmental assessment?<br>• Is there anything you would like to add towards the end?<br><br>Thank you so much for your time and your contribution.
