# Supplementary material for: Increased family psychosocial focus during children’s developmental assessments: a study of parents’ views
Source: BMC Pediatr. 2024 May 15;24:335. doi: 10.1186/s12887-024-04800-4 (PMC11094963; doi:10.1186/s12887-024-04800-4)
Supplement: Supplementary file 2 — Supplementary Material 2 [file 12887_2024_4800_MOESM2_ESM.pdf]

Additional file 2: example of a structured child record

5 week preventive child health examination

Date: **Birth date:** **Examination by:** Doctor ☐ Midwife ☐ Nurse ☐

Mother's name: **Child's name:**

Who is accompanies the child:

The birth

Birth weight: ……………………….. Born in week: ………………………..

Birth complications: yes ☐ no ☐ Comment: ………………………………………………………

Neonatal complications: yes ☐ no ☐ Comment: ………………………………………………………

Conversation with the parents about the pregnancy, birth and new-born period: yes ☐ no ☐

Comment: ………

Family and social conditions

Parents living together: yes ☐ no ☐ Comment: ………………………………………………………

Parents healthy: yes ☐ no ☐ Comment: ………………………………………………………

Conversation about mental well-being: yes ☐ no ☐

The mother, problems: yes ☐ no ☐ Comment: ………………………………………………………

The father, problems: yes ☐ no ☐ Comment: ………………………………………………………

No. siblings: ….. , healthy yes ☐ no ☐ Comment: ………………………………………………………

Conversation about social conditions: (parents' jobs, economy, accommodation, social benefits) yes ☐ no ☐

Comment: ………

Relationships with grandparents: yes ☐ no ☐ Elaborate: ………………………………………………………

………

Conversation about network: (relation to siblings, family, friends, colleagues, neighbours) yes ☐ no ☐

Comment: ………

………

Current

Regulation of the child:

Problems with breastfeeding: yes ☐ no ☐ Comment: ………………………………………………………

Problems with sleep: yes ☐ no ☐ Comment: ………………………………………………………

Problems with circadian rhythm: yes ☐ no ☐ Comment: ………………………………………………………

Problems with crying: yes ☐ no ☐ Comment: ………………………………………………………

Illness/health issues since birth: yes ☐ no ☐ Comment: ………………………………………………………

Do the mother/parents experience problems in their care of the child?

yes ☐ no ☐ Comment: ………

Contact with the health nurse: yes ☐ no ☐ Comment: …………………………………………

Health examination

Head measurement: ………….. Weight: ………….. Length: ………….. Weight gain pr. Week: …………..
(100-400 g +/- for both boys and girls)

Observation of collaboration, autonomy and responsiveness:

Mother-child:

Did you observe?	Yes /no	Positive	Negative
Cooperation			
Autonomy			
Responsiveness			

Comments: ………………………………………………

……………………………………………………………………

Father-child (or other companion):

Did you observe?	Yes /no	Positive	Negative
Cooperation			
Autonomy			
Responsiveness			

Comments: …………………………………………………

……………………………………………………………………

Was something worrying? ……………………………………………………………………………………………………

Physical examination:

Normal interaction with the child: yes ☐ no ☐ Comment: …………………………………………………
(The child listens and fixates its eyes briefly) ……………………………………………………………

Overall normal assessment: yes ☐ no ☐ Comment: …………………………………………………
(Casual, comfortable with calm, symmetrical movements.
Reflexes, tonus. Lifting the head from abdominal position.
Crying with variation) ……………………………………………………………………

Normal eye pupils, red eye reflex: yes ☐ no ☐ Comment: …………………………………………………

Normal skull, mouth, skin, neck: yes ☐ no ☐ Comment: …………………………………………………

Normal heart/lung stethoscope: yes ☐ no ☐ Comment: …………………………………………………

Normal hip joints: yes ☐ no ☐ Comment: …………………………………………………

Normal genitals: yes ☐ no ☐ Comment: …………………………………………………

Additional info: ………

Overall assessment

Short resume of important findings:

………

………

………

Follow up before next preventive child health examination:

Consultation-control ☐ Home visiting nurse ☐ Specialist/hospital ☐

Social services ☐ Other effort ☐ No extra effort needed ☐

Comment: ……

………
