# Supplementary figures and images for: Increased family psychosocial focus during children’s developmental assessments: a study of parents’ views
Source: BMC Pediatr. 2024 May 15;24:335. doi: 10.1186/s12887-024-04800-4 (PMC11094963; doi:10.1186/s12887-024-04800-4)

## Additional file 3: coding tree

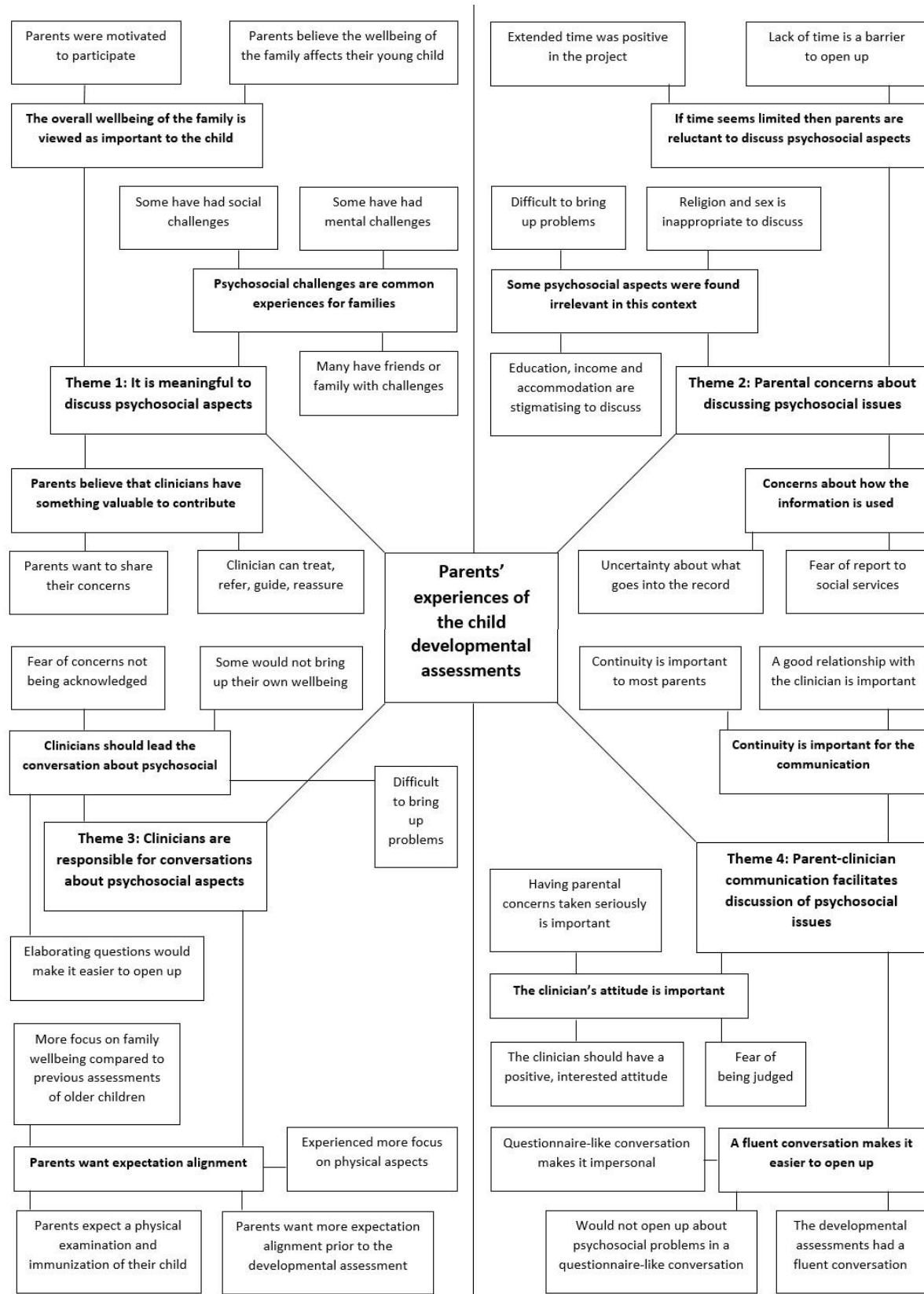

Supplement: Supplementary file 3 — Supplementary Material 3 [file 12887_2024_4800_MOESM3_ESM.pdf]
